# Impaired Postural Control in Healthy Men at Moderate Altitude (1630 M and 2590 M): Data from a Randomized Trial

**DOI:** 10.1371/journal.pone.0116695

**Published:** 2015-02-27

**Authors:** Katrin Stadelmann, Tsogyal D. Latshang, Christian M. Lo Cascio, Ross A. Clark, Reto Huber, Malcolm Kohler, Peter Achermann, Konrad E. Bloch

**Affiliations:** 1 Institute of Pharmacology and Toxicology, University of Zurich, Zurich, Switzerland; 2 Zurich Center for Integrative Human Physiology, University of Zurich, Zurich, Switzerland; 3 Pulmonary Division, University Hospital Zurich, Zurich, Switzerland; 4 University Children’s Hospital Zurich, Zurich, Switzerland; 5 Department of Physiotherapy, Faculty of Medicine, Dentistry and Health Sciences, The University of Melbourne, Melbourne, Australia; Johns Hopkins University SOM, UNITED STATES

## Abstract

**Objectives:**

Intact postural control is essential for safe performance of mountain sports, operation of machinery at altitude, and for piloting airplanes. We tested whether exposure to hypobaric hypoxia at moderate altitude impairs the static postural control of healthy subjects.

**Methods:**

In 51 healthy men, median age 24 y (quartiles 20;28), static control was evaluated on a balance platform in Zurich, 490 m, and during a 4-day sojourn in Swiss mountain villages at 1630 m and 2590 m, 2 days each. The order of altitude exposure was randomized. Total center of pressure path length (COPL) and sway amplitude measured in two directions by a balance platform, and pulse oximetry were recorded. Data were compared between altitudes.

**Results:**

Median (quartiles) COPL during standing on both legs with eyes open at 490 m and in the evenings on the first and second days at 1630 and 2590 m, respectively were: 50 (45;57), 55 (48;62), 56 (49;61), 53 (47;59), 54 (48;60) cm, P<0.001 ANOVA. Corresponding arterial oxygen saturation was 97% (96;97), 95% (94;96), 95%(94;96), 92%(90;93), 93%(91;93), P<0.001. Anterior-posterior sway amplitudes were larger at 1630 and 2590 m compared to 490 m, P<0.001. Multiple logistic regression analysis confirmed that higher altitudes (1630 and 2590m) were independently associated with increased COPL when controlled for the order of altitude exposure and age (P=0.001).

**Conclusions:**

Exposure to 1630 and 2590m was associated with impaired static postural control even when visual references were available.

**Trial Registration:**

ClinicalTrials.gov NCT01130948.

## Introduction

Control of posture is essential for the safe performance of many activities of daily life, and in particular for sports, operating cars, machinery and for piloting airplanes. Furthermore, poor postural control is a major contributing factor to an increased risk for falls in the elderly [1]. Altitude travel for professional and leisure activities is increasingly common among millions of lowlanders worldwide [2,3]. As the central nervous system is sensitive to hypoxia, ascent to altitude may lead to impaired cognitive performance and motor control [4,5]. Dizziness, mental and muscle fatigue, and decrements in alertness and psychomotor performance have been shown to occur in hypoxic environments at high altitude (> 4000 m) [6]. Furthermore, impairments in postural stability during short exposures of a few hours to simulated altitudes between 1500 m and 5500 m have been reported [7–9]. However, it remains unclear whether such impairments persist or even aggravate during a prolonged stay at altitude, and to which extent they are altitude-dependent [7,9,10]. Baumgartner et al. [11] did not observe an improvement of posturographic performance in healthy mountaineers evaluated over the course of 3 days at the Capanna Regina Margherita research station at 4559 m. Whether similar impairments in postural control occur and persist during a prolonged stay even at moderate altitudes of 1600 to 2600 m remains unknown. Since most mountain resorts with frequent tourism are located at such moderate elevations more knowledge on this issue is highly desirable and relevant. Therefore, the purpose of the current study was to investigate static postural control in healthy subjects at 490 m and during a four-day sojourn at an alpine resort at moderate altitude. Applying a randomized cross-over study design we tested the hypothesis that static control was impaired in an altitude dependent manner at Davos Jakobshorn (2590 m) compared to Davos Wolfgang (1630 m) and Zurich (490 m), respectively.

## Materials and Methods

The protocol for this trial and supporting CONSORT checklist are available as supporting information; see S1 CONSORT Checklist and S1 Protocol.

### Subjects

Fifty-one healthy male volunteers, mean age ± SD: 26.9 ± 9.3 years (range: 20–67), were recruited. Subjects were accepted only if they were in good health, taking no medications regularly, had no history of altitude related illness during previous stays at < 2500 m, had not travelled to altitudes > 1500 m in the two weeks prior to the study. The study protocol was approved by the ethical committee of the Canton of Zurich (Switzerland), and participants gave their written informed consent.

### Protocol and interventions

**Fig 1:**
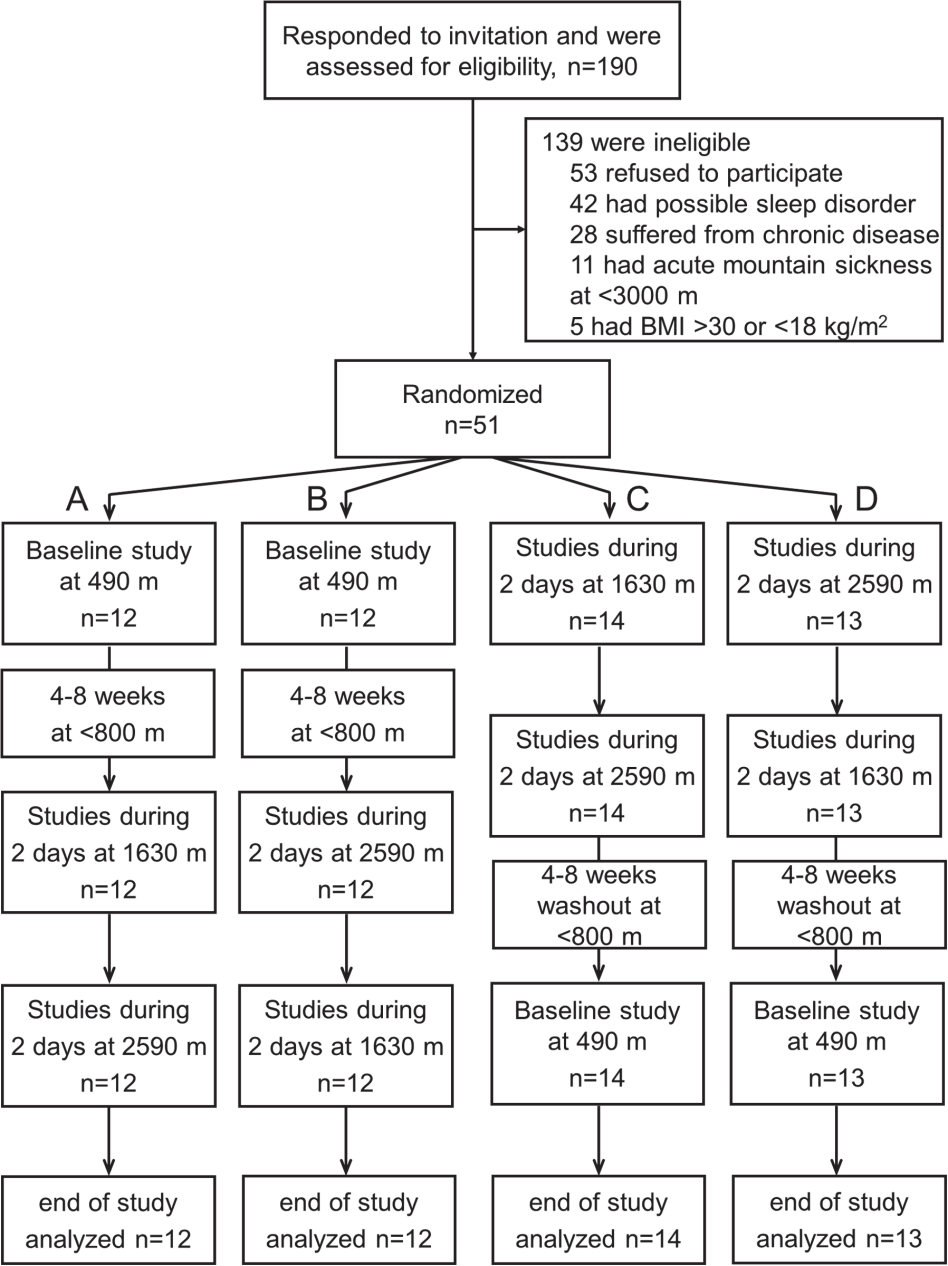
Consort flow chart. 51 participants were randomized to 4 differend schedules of altitude exposure. The flow chart is the same as that for the study reported in [12].

This study was part of a randomized cross-over trial (www.ClinicalTrials.gov, NCT01130948) evaluating the effects of altitude exposure on various physiologic outcomes. Data on the effects of altitude on sleep and cardiovascular function are described elsewhere [12–14], including the consort flow chart (Fig. 1). The data on static postural control, the topic of the current paper, have not been published.

Measurements were performed in the period from July to October 2010 during one day at the University Hospital of Zurich (baseline, 490 m, 1608 ft, barometric pressure [PB] 719 Torr), and for 4 days at two study locations in the Swiss Alps, i.e. for 2 days at Davos Wolfang (1630 m, 5348 ft, PB 630 Torr), and 2 days at Davos Jakobshorn (2590 m, 8497 ft, PB 562 Torr). The order of altitude exposure was randomized with a balanced design by letting participants select a study time slot according to their preference and availability without being aware of the corresponding altitude exposure sequence. Each time slot corresponded to one of the following 4 sequences of altitude exposure (Fig. 1): A) 490 m—1630 m—1630 m—2590 m—2590 m; B) 490 m—2590 m—2590 m-1630 m—1630 m; C) 1630 m—1630 m—2590 m—2590 m—490m; D) 2590 m—2590 m—1630 m—1630 m—490 m. A wash-out phase of 2–4 weeks was interposed between studies at altitude and at 490 m and vice versa. Subjects traveled from Zurich to the altitude locations by train and cable car. On study days at 490 m and during the stay at altitude subjects had to avoid strenuous exercise and to stay at the same altitude and within 200 m of the study location. The nocturnal rest period was from 23:00 to 06:00. Standing balance was measured between 5 and 6 pm and between 10 and 11 am.

### Standing balance

Standing balance was measured with a rectangular balance board 30x50cm in size, containing four transducers to assess movements of the center of pressure (COP; Wii Balance Board, Redmond, WA, USA). The balance board was interfaced with a computer using specialized software as described previously [15]. Repeated calibrations with known weights were performed. Measurements of the standing balance by the balance board have been extensively validated [15].

Standing balance was assessed in the following three conditions: 1) standing on both legs, 2) on the right leg, and 3) on the left leg. Each condition was repeated 3 times with eyes open and then with eyes closed resulting in a total of 18 tests per session (see Table 1). Measurements on both legs lasted 30 s, and measurements on one leg 10 s, with breaks of at least 20 s between tests. The balance board was positioned 1.5 m in front of a wall. During eyes open tests subjects were instructed to focus on a black dot, 2 cm in diameter, fixed at the wall at the height of the subject’s eyes, and to keep their arms parallel to the body during all measurements. Standing balance on both legs was performed with a 30° angle between feet and with heels 2 cm apart [16]. For assessment of standing balance on one leg with the foot positioned in the center of the balance board, subjects had to rise the other leg to a 90° angle in the hip and bend the knee to a 90° angle so that the lower leg was in vertical position [17].

**Table 1:** Test protocol.

Test number	Condition			Duration [s]	Eyes
	Both legs	Right leg	Left leg		
1	X			30	open
2		X		10	open
3			X	10	open
4	X			30	open
5		X		10	open
6			X	10	open
7	X			30	open
8		X		10	open
9			X	10	open
10	X			30	closed
11		X		10	closed
12			X	10	closed
13	X			30	closed
14		X		10	closed
15			X	10	closed
16	X			30	closed
17		X		10	closed
18			X	10	closed

### Oxygen saturation

Oxygen saturation (SpO2) was measured by pulsoximetry every morning and evening while subjects were resting quietly in supine position.

### Data analysis

**Fig 2:**
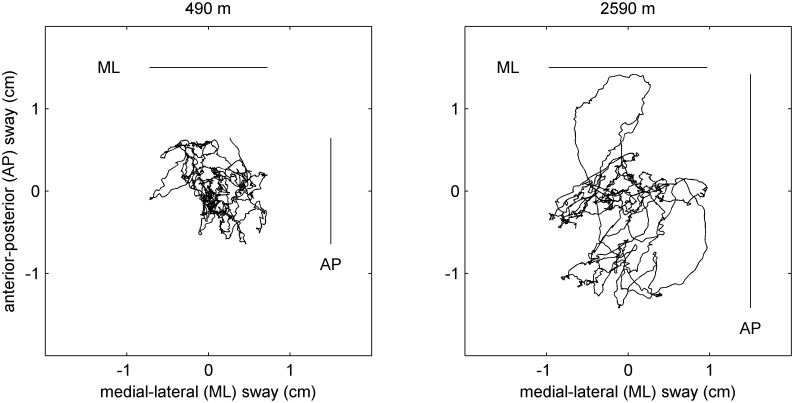
Center of pressure recorded in an individual during a 30 s balance board test on both legs with eyes open at 490 m and 2590 m. Center of pressure path length (COPL) = 45 and 56 cm; anterior-posterior (AP) sway amplitude = 1.3 and 2.8 cm; medial-lateral (ML) sway amplitude = 1.4 and 1.9 cm.

Three outcome measures were derived from each balance test: the pathway length of the COP movement; the movement amplitude in anterior-posterior (AP) direction; and the movement amplitude in medial-lateral (ML) direction (Fig. 2). Means of the three repeated measurements in each condition and with eyes open and eyes closed, respectively, were computed and the results used for further analysis.

Data were summarized by means (SD) and medians (quartiles) for normal and non-normal distribution, respectively. For statistical analysis, data were transformed to obtain a normal distribution where appropriate. Path length was transformed to 1/square root (path length). The anterior-posterior and medial-lateral amplitude were log transformed.

A mixed model ANOVA was performed to investigate differences between the five test days at the different altitudes (i.e., one at 490 m, two at 1630 m and two at 2590 m). Post-hoc Wilcoxon signed ranks tests with Bonferroni correction were performed comparing results from different altitudes whenever the overall ANOVA was significant (p<0.05). In order to evaluate the effect of altitude on static control while controlling for potential confounders, univariate and multivariate ordinal logistic regression analysis was employed with COP path length, antero-posterior and medial-lateral sway, respectively, as dependent variables after transformation to quintiles. Independent variables in this analysis were altitude (490, 1630, 2590 m), oxygen saturation (%), altitude exposure sequence (coded 1 to 4), consecutive number of test days (1 to 5), evening and morning tests (coded 1, 2), eyes open and closed (coded 1 and 2), and age (years). Altitude and age, and all other independent variables with p<0.1 in univariate analysis were entered into the multivariate analysis. Sample size estimations indicating a minimal required number of 50 participants were based on the outcomes apnea/hypopnea index and psychomotor vigilance response reported previously [12]. A probability of P<0.05 was considered statistically significant.

## Results

**Fig 3:**
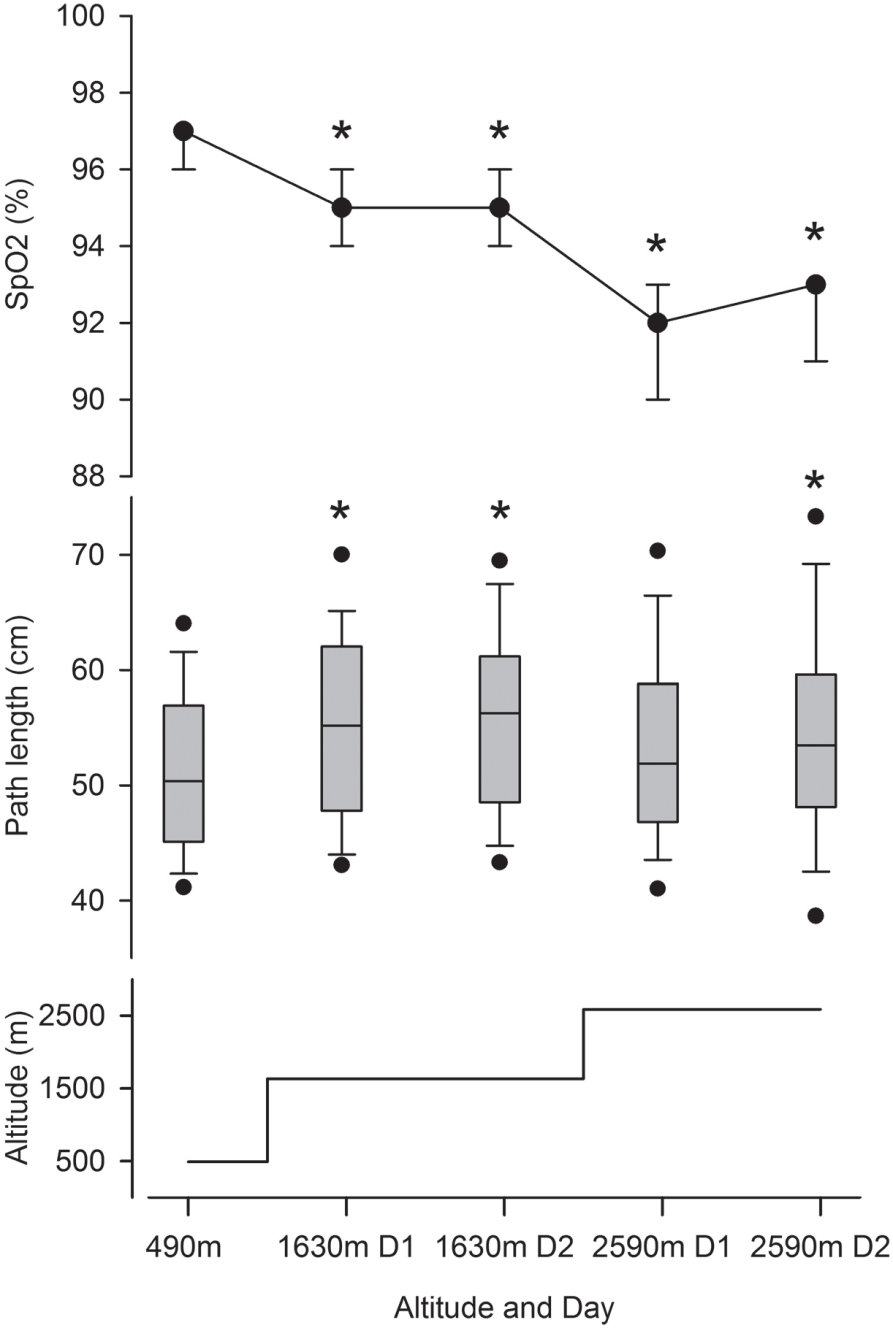
Results from evening measurements of arterial oxygen saturation by pulse oximetry (SpO2, medians and quartiles, top panel), and center of pressure path length by the balance board (middle panel) in the 51 study participants standing on both legs with eyes open along with altitude profile. Box plots show medians, quartiles, and whiskers representing the 10^th^ and 90^th^ percentiles, and dots the 5^th^ and 95^th^ percentiles. * p<0.05 vs. 490 m. D1 and D2 = 1^st^ and 2^nd^ day at corresponding altitude.

Fig. 1 shows the participant flow. An example of a center of pressure path length (COPL) recording at 490 m and at 2590 m with a large path length is illustrated in Fig. 2. Tables 2 and 3 summarize the COPL and the anterior-posterior sway amplitude at the different altitudes along with oxygen saturation for tests on both legs. S1 and S2 Tables show corresponding values for single leg tests. The COPL during balance tests with eyes open on both legs, right leg, and left leg was longer at the higher altitudes compared to 490 m (Table 2, S1 Table) and this was observed in both morning and evening measurements. The findings from evening tests on both legs are summarized in Fig. 3. In contrast, the COPL of measurements with eyes closed was not consistently changed with increasing altitude although the path lengths were longer than corresponding values with eyes open.

**Table 2:** Center of pressure path length at different altitudes.

Measurement	Time	Eyes	490 m	1630 m Day 1	1630 m Day 2	2590 m Day 1	2590 mDay 2	P ANOVA overall
**Both legs**	evening	open	50 [45, 57]	55 [48, 62] *	56 [49, 61] *	52 [47, 59]	54 [48, 60] *	<0.001
**Both legs**	evening	closed	62 [55, 71]	65 [57, 76]	64 [57, 76]	64 [56, 76]	63 [58, 75]	0.150
**Both legs**	morning	open	52 [47, 58]	54 [49, 63] *	56 [49, 60] *	55 [47, 61]	55 [48, 60] *	0.003
**Both legs**	morning	closed	61 [57, 69]	65 [60, 74]	63 [57, 69]	65 [58, 78]	68 [59, 73]	0.042
**Pulse oximetry, %**	evening	open	97 [96, 97]	95 [94, 96] *	95 [94, 96] *	92 [90, 93] *	93 [91, 93] *	<0.001
**Pulse oximetry, %**	morning	open	97 [97, 98]	96 [95, 96] *	96 [95, 96] *	93 [92, 94] * ^¶^	94 [93, 94] * ^¶^	<0.001

Data are presented as median path length in cm [25^th^, 75^th^ percentile].

P ANOVA overall: Mixed model ANOVA with factor condition (490 m, 1630 m day 1, 1630 m day 2, 2590 m day 1, 2590 m day 2).

* p<0.0125 compared to 490 m (Bonferroni correction), post-hoc Wilcoxon signed ranks test.

^¶^ p<0.0125 compared to 1650 m, day 1 and 2 (Bonferroni correction), post-hoc Wilcoxon signed ranks test.

**Table 3:** Anterior-posterior sway amplitude at different altitudes.

Measurement	Time	Eyes	490 m	1630 m Day 1	1630 m Day 2	2590 m Day 1	2590 m Day 2	P ANOVA Overall
**Both legs**	evening	open	1.9 [1.7, 2.4]	2.2 [2.0, 2.6] *	2.3 [2.1, 2.8] *	2.1 [1.7, 2.4]	2.1 [1.7, 2.7]	<0.001
**Both legs**	evening	closed	2.8 [2.3, 3.3]	2.9 [2.5, 3.4]	2.7 [2.3, 3.2]	2.7 [2.1, 3.4]	2.8 [2.3, 3.4]	0.480
**Both legs**	morning	open	2.3 [1.9, 2.8]	2.5 [2.0, 3.0]	2.4 [2.1, 3.0]	2.3 [1.8, 3.0]	2.3 [1.9, 2.8]	0.099
**Both legs**	morning	closed	2.8 [2.4, 3.4]	3.0 [2.5, 3.5]	2.8 [2.4, 3.5]	3.2 [2.4, 3.5]	3.0 [2.5, 3.5]	0.366

Data are presented as median sway ampltitude in cm [25^th^, 75^th^ percentile]. Oxygen saturation values are the same as in Table 2.

P ANOVA overall: Mixed model ANOVA with factor condition (490 m, 1630 m day 1, 1630 m day 2, 2590 m day 1, 2590 m day 2).

* p<0.0125 vs. to 490 m (Bonferroni correction), post-hoc Wilcoxon signed ranks.

Consistent with the longer COPL, the anterior-posterior sway amplitude was also increased at 1630 m during measurements in the eyes open condition with the exception of standing balance on both legs in the morning (Table 3). Furthermore, standing balance on the right and left leg with eyes open also showed significant changes on the medial-lateral sway amplitude on morning and evening measurements (S3 Table).

Individual changes in path length at altitude are illustrated in S1 Fig. Seventy-seven and 69% of the subjects showed an increase in path length at 1630 m and 2590 m, respectively for measurements on both legs with eyes open. Seventy-eight and 82% showed a reduction in balance performance for the measurements on the left and right leg respectively at 1630 m. Changes in path length on one leg at 2590 m were not significant. Anterior-posterior and medial-lateral sway amplitudes were both increased at 1630 m, but remained unchanged at 2590 m (Table 3 and S3 Table).

Multivariate ordinal logistic regression analysis confirmed an independent effect of altitude on the COPL (Table 4) even when controlled for potential confounders. Thus, the odds-ratio that the COPL increased by one quintile with ascent from 490 to 1630 m, and with ascent from 490 to 2590 m, respectively, was 1.7 (Table 4). Performing tests with eyes closed was a strong predictor of a larger path length (odds-ratio 7.1, Table 4). Multivariate logistic regression analysis further revealed an independent effect of altitude on the anterior-posterior sway amplitude at 1630 m (Table 5). When ordinal regression analysis was performed with data from both altitudes (1650 and 2590 m) vs. 490 m baseline, similar results as those with all 3 altitudes entered separately were observed. The odds ratio for an increase in COPL by one quintile with ascent from 490 m to altitude (1650 and 2590 m) was 1.70 (95% confidence limits 1.29 to 2.53, P <0.001); the corresponding odds ratio for an increase in antero-posterior sway amplitude was 1.31 (95% CI 0.97 to 1.79, P = 0.074). There was no association of altitude with medial-lateral sway amplitude (S4 Table).

**Table 4:** Effect of altitude on center of pressure path length: ordinal logistic regression analysis.

	Univariate			Multivariate		
Dependent variable: quintiles of center of pressure path length	Odds ratio	95% CI	P	Odds ratio	95% CI	P
Altitude						
1630 vs. 490 m	1.55	1.22 to 1.97	<0.001	1.72	1.27 to 2.34	<0.001
2590 vs. 490 m	1.53	1.19 to 1.96	0.001	1.73	1.25 to 2.39	0.001
SpO2, %	0.95	0.89 to 1.02	0.164			
Altitude exposure sequence						
2 vs. 1	0.51	0.22 to 1.21	0.129	0.43	0.15 to 1.22	0.113
3 vs. 1	0.34	0.12 to 0.95	0.040	0.23	0.06 to 0.86	0.029
4 vs. 1	0.45	0.21 to 0.10	0.049	0.40	0.15 to 1.03	0.057
Consecutive number of test days	1.02	0.95 to 1.10	0.596			
Eyes closed vs. eyes open	6.27	4.43 to 8.87	<0.001	7.12	4.82 to 10.50	<0.001
Morning vs. evening test	1.09	0.96 to 1.23	0.176			
Age, y	1.00	0.96 to 1.03	0.710	0.98	0.95 to 1.02	0.394

Univariate and multivariate logistic regressions were performed on quintiles of COP path lengths with an odds ratio of 1 corresponding to the lowest quintile. Altitude, age, and all variables with P<0.1 in univariate analysis were entered into the multivariate model. Altitude exposure sequence was: 1 = 2590-1630-490 m; 2 = 1630-2590-490m; 3 = 1630-2590-490 m; 4 = 2590-1630-490 m; consecutive number of test days was 1 to 5; eyes open and closed were coded as 1 and 2, respectively; morning and evening tests were coded as 1 and 2, respectively. CI = confidence interval.

**Table 5:** Effect of altitude on anterior-posterior sway: ordinal logistic regression analysis.

	Univariate			Multivariate			
Dependent variable: quintiles of anterior-posterior sway	Odds ratio	95% CI	P	Odds	95% CI	P
Altitude						
1630 vs. 490 m	1.35	1.04 to 1.75	0.023	1.46	1.09 to 1.96	0.012
2590 vs. 490 m	1.11	0.80 to 1.53	0.543	1.15	0.83 to 1.64	0.449
SpO2, %	1.01	0.94 to 1.09	0.838			
Altitude exposure sequence						
2 vs. 1	0.59	0.26 to 1.33	0.204			
3 vs. 1	0.81	0.36 to 1.80	0.605			
4 vs. 1	0.58	0.27 to 1.24	0.162			
Consecutive number of test days	0.92	0.85 to 1.00	0.058	0.91	0.83 to 0.98	0.045
Eyes closed vs. eyes open	4.49	3.24 to 6.23	<0.001	4.62	3.28 to 6.52	<0.001
Morning vs. evening test	1.63	1.41 to 1.88	<0.001	1.68	1.43 to 1.98	<0.001
Age, y	1.00	0.96 to 1.04	0.922	1.00	0.95 to 1.04	0.883

Univariate and multivariate logistic regressions were performed on quintiles of values of the anterior-posterior sway amplitude with an odds ratio of 1 corresponding to the lowest quintile. Altitude, age, and all variables with P<0.100 in univariate analysis were entered into the multivariate model. Altitude exposure sequence was: 1 = 2590-1630-490 m; 2 = 1630-2590-490m; 3 = 1630-2590-490 m; 4 = 2590-1630-490 m; consecutive number of test days was 1 to 5; eyes open and closed were coded as 1 and 2, respectively; morning and evening tests were coded as 1 and 2, respectively. CI = confidence interval.

## Discussion

We studied the effect of a stay at moderate altitude for four days on static control in a large cohort of healthy men. The results of our randomized cross-over study demonstrate that the center of pressure (COPL) and the anterior-posterior sway amplitude were increased at moderate altitude which is consistent with an impaired static control at 1630 m and 2590 m compared to 490 m. The findings may be relevant for a large number of persons traveling to moderate altitude.

This is the first field study to examine changes in balance performance at two moderate altitudes (1630 m and 2590 m) compared to low altitude (490 m). Compared to investigations at simulated altitudes, the results from the current four-day study performed at two real moderate altitudes represent the environmental conditions of many mountain tourists worldwide. They are also relevant for persons operating machinery at altitude, as well as for aircrew members, as a cabin pressure equivalent to up to 2430 m (8000 ft) is reached in commercial airplanes [18]. Our results show that the COPL and the anterior-posterior sway amplitude are affected already at 1630 m. Thus, static postural control was impaired at a relatively low altitude. These observations corroborate and extend data from previous studies demonstrating impaired static postural control at simulated higher altitudes and with shorter exposures of a few minutes to 24 h [8,9,19]. In line with these findings Holness et al. [8] observed an increase in anterior-posterior sway in seven healthy men if oxygen saturation was reduced to 70% by letting them breathe a normobaric hypoxic gas mixture. Moreover, Cymerman et al. [19] reported worsening of postural stability in 19 subjects during measurements on a dynamic platform balance system at an altitude equivalent to 4300 m simulated in a decompression chamber, and Nordahl et al. [9] observed an increase in body sway in 16 military aircrew members standing on a balance platform at simulated altitudes of 5500 m, 4300 m and 2400 m.

Consistent with previous studies that reported alterations mainly or exclusively in the anterior-posterior direction during altitude exposure [8,9,11] we observed an increase predominantly in the anterior-posterior sway amplitude (Table 3) and, to a lesser extent, in medial-lateral sway amplitude (S3 Table, online supplement). However, the latter was only affected when standing on one leg, and not when standing on both legs, most likely because postural control gets more challenging when holding balance on one leg. In addition, our larger sample size provided a greater power to detect minor changes. In contrast to the medial-lateral sway amplitude, the sway amplitude in the anterior-posterior plane was increased in all three conditions (right leg, left leg and both legs) at moderate altitude. These findings therefore support prior assumptions, that control over movements in the anterior-posterior plane is most sensitive to hypobaric hypoxia at altitude [8,9,11]. We found larger COPL and sway amplitudes with eyes closed than with eyes open (Tables 2 and 3). Vision is an important sensory input for postural control along with the proprioceptive and vestibular feedback [20]. Related to that and in agreement with Holness et al. [8], we observed that, independent of altitude, performing tests with eyes closed was a strong predictor of a large path length. The dominant effect of withdrawing visual references by closing the eyes (and the large individual variability of this effect) has concealed the more moderate effect of altitude in univariate analyses (Tables 2 and 3). When the effects of closing the eyes and other confounders were controlled for by multivariate analyses, significant, independent effects of altitude on COPL and sway amplitude were confirmed (Tables 4 and 5).

In the current altitude field study the variables representing postural control indicated impairment at 1630 and 2590 m compared to 490 m, respectively, but the differences between 1630 and 2590 m were not statistically significant (Tables 2 and 3). It remains elusive whether the lack of further impairment with ascent from 1630 to 2590 m is due to compensatory mechanisms such as an increase in cerebral perfusion or other unknown mechanisms. Although the multiple regression analysis revealed a significant effect of altitude on postural control, SpO2 was not independently associated with measures of postural control (Table 4). This might be related to the flat shape of the oxygen-hemoglobin dissociation curve as the arterial oxygen partial pressures corresponding to 1630 and 2590 m resulting in poor sensitivity of pulse oximetry to subtle changes in arterial PO2. Consistent with the study of Baumgartner et al. [12], who reported that posturographic parameters remained impaired over the course of three consecutive days at 4559 m, we did not find an effect of the number of consecutive tests at altitude on COPL (Table 4). We therefore conclude that in regard to COPL, no acclimatization or learning effect was detectable during the four days at altitude. Correspondingly, as reported in our previous paper [13], the participants of the current study did not reveal significant changes in the mean nocturnal oxygen saturation and end-tidal PCO2 between night 1 and 2 at 1630 m, and an increase in mean nocturnal oxygen saturation of only 1% from night 1 to night 2 at 2590 m.

Although we found that participants in the current study experienced impairments in postural control during their stay at altitude we failed to demonstrate a cognitive impairment (data reported in [12]). Studies have shown that during exposure to hypoxia oxygen delivery to the brainstem and cerebellum, areas involved in static control, is better defended than oxygen delivery to cortical regions involved in cognitive processing [21]. Our findings are therefore more likely explaind by insufficient sensitivity of the cognitive tests than by a greater vulnerability to hypoxia of brain regions involved static control compared to regions involved in cognition.

Since our study included predominantly young, male subjects the conclusions may not necessarily apply to older or female individuals or to patients with cardiorespiratory or neurologic diseases.

In conclusion, this is the first field study investigating postural control over the course of several days at moderate altitudes corresponding to an elevation of many tourist destinations worldwide. Postural control was impaired already at 1630 m while several cognitive tests performed in the same subjects at 1630 m and 2590 m were not affected [12]. Since optimal postural control is essential for many activities of daily living, and poor postural control contributes to the risk of falls in the elderly [1], the findings of the current study may have major implications for many persons exposed to mild hypobaric hypoxia at moderate altitude.

## Supporting Information

S1 CONSORT Checklist (DOC)

S1 Data (ZIP)

S1 Fig Individual changes in Center of Pressure Path Length (COPL) with ascent to altitude. The difference in COPL [cm] recorded at 1630 m and 2590 m, respectively, minus the corresponding value at 490 m is plotted for each subject (n = 51) at 1630 m and 2590 m (mean values of day 1 and 2 at altitude and of morning and evening measurements are illustrated). The bars representing individual values of the difference in COPL are sorted by size. Negative values indicate an improvement and positive values a decrease of postural control at altitude. The vertical line indicates the transition from negative to positive values. P values are indicated for comparisons (Wilcoxon signed ranks test) of median values at moderate altitude (morning, evening and day1, day2) with corresponding medians at 490 m. ns = not significant.
(TIF)

S1 Protocol Trial Protocol. (PDF)

S1 Table Center of pressure path length at different altitudes, single leg tests. Summary statistics for the results obtained during measurements on the right and left leg, with eyes open and closed, and in the evening and morning, respectively.
(DOCX)

S2 Table Anterior-posterior sway amplitude at different altitudes, single leg tests. Summary statistics for the results obtained during measurements on the right and left leg, with eyes open and closed, and in the evening and morning, respectively.
(DOCX)

S3 Table Medial-lateral sway amplitude at different altitudes. Summary statistics for the results obtained during measurements on both legs, the right and left leg, with eyes open and closed, and in the evening and morning, respectively.
(DOCX)

S4 Table Effect of altitude on medial-lateral sway: ordinal logistic regression analysis. Results of univariate and multivariate logistic regression analysis performed on medial-lateral sway amplitude with altitude, pulse oximetry, altitude exposure sequence, consecutive number of tests, eyes open/closed, evening/morning, and age as independent variables.
(DOCX)
