# Supplementary figures and images for: Impaired Postural Control in Healthy Men at Moderate Altitude (1630 M and 2590 M): Data from a Randomized Trial
Source: PLoS One. 2015 Feb 27;10(2):e0116695. doi: 10.1371/journal.pone.0116695 (PMC4344242; doi:10.1371/journal.pone.0116695)

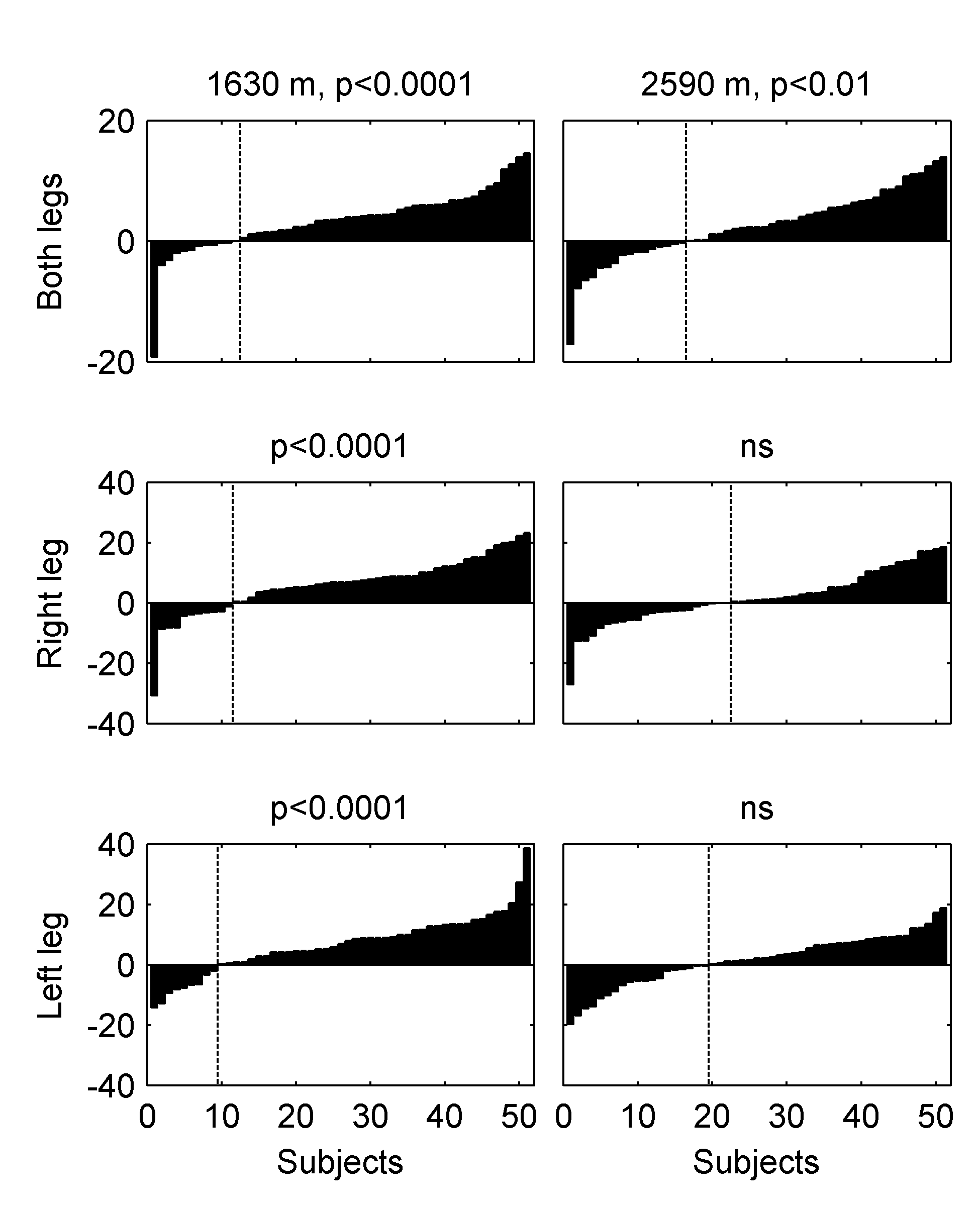

Supplement: S1 Fig — The difference in COPL [cm] recorded at 1630 m and 2590 m, respectively, minus the corresponding value at 490 m is plotted for each subject (n = 51) at 1630 m and 2590 m (mean values of day 1 and 2 at altitude and of morning and evening measurements are illustrated). The bars representing individual values of the difference in COPL are sorted by size. Negative values indicate an improvement and positive values a decrease of postural control at altitude. The vertical line indicates the transition from negative to positive values. P values are indicated for comparisons (Wilcoxon signed ranks test) of median values at moderate altitude (morning, evening and day1, day2) with corresponding medians at 490 m. ns = not significant. (TIF) [file pone.0116695.s003.tif]
