# Supplementary material for: Impaired Postural Control in Healthy Men at Moderate Altitude (1630 M and 2590 M): Data from a Randomized Trial
Source: PLoS One. 2015 Feb 27;10(2):e0116695. doi: 10.1371/journal.pone.0116695 (PMC4344242; doi:10.1371/journal.pone.0116695)
Supplement: S1 Table — Summary statistics for the results obtained during measurements on the right and left leg, with eyes open and closed, and in the evening and morning, respectively. (DOCX) [file pone.0116695.s005.docx]

**Impaired postural control in healthy men at moderate altitude (1630 m and 2590 m). Data from a randomized trial.**

K. Stadelmann^1,2^, T. D. Latshang^3^, C. M. Lo Cascio^3^, R. A. Clark^5^, R. Huber^2,4^, M. Kohler^2,3^, P. Achermann^1,2^* and K. E. Bloch^2,3^*

**Table S1. Center of pressure path length at different altitudes, single leg tests**

| **Measurement** | **Time** | **Eyes** | **490 m** | **1630 m Day 1** | **1630 m Day 2** | **2590 m Day 1** | **2590 m Day 2** | **P ANOVA Overall** |
| --- | --- | --- | --- | --- | --- | --- | --- | --- |
| **Right leg** | evening | open | 42  [37, 53] | 52 [47, 64] * | 49 [42, 63] * | 47 [40, 60] | 44 [38, 60] | <0.001 |
| **Left leg** | evening | open | 42 [36, 57] | 52 [44, 66] * | 48.4 [42, 60] * | 47 [39, 57] | 45 [35, 56] | <0.001 |
| **Right leg** | evening | closed | 118 [95, 145] | 111 [94, 148] | 106 [91, 143] | 111 [93, 133] | 110 [91, 138] | 0.819 |
| **Left leg** | evening | closed | 108 [93,134] | 113 [93,135] | 110 [89,131] | 116 [94, 136] | 107 [91, 138] | 0.557 |
| **Right leg** | morning | open | 45 [39, 53] | 50 [41, 57] * | 49[41, 60] * | 46.0 [39, 59] | 44 [36, 59] | <0.001 |
| **Left leg** | morning | open | 42 [36, 53] | 48 [43, 61] * | 48 [41, 57] * | 46 [38, 57] | 45 [38, 56] | <0.001 |
| **Right leg** | morning | closed | 112 [88, 147] | 112 [89, 139] | 111 [93, 132] | 116 [98, 147] | 117 [97, 145] | 0.067 |
| **Left leg** | morning | closed | 111 [90, 143] | 109 [90, 133] | 110 [96, 123] | 115 [95, 136] | 111 [94, 140] | 0.168 |

Data are presented as median path length in cm [25^th^, 75^th^ percentile]. Oxygen saturation values are the same as in table 2 of the main paper.

P ANOVA overall: Mixed model ANOVA with factor condition (490 m, 1630 m day 1, 1630 m day 2, 2590 m day 1, 2590 m day 2).

* p<0.0125 compared to 490 m (Bonferroni correction), post-hoc Wilcoxon signed ranks test.
