# Supplementary material for: Impaired Postural Control in Healthy Men at Moderate Altitude (1630 M and 2590 M): Data from a Randomized Trial
Source: PLoS One. 2015 Feb 27;10(2):e0116695. doi: 10.1371/journal.pone.0116695 (PMC4344242; doi:10.1371/journal.pone.0116695)
Supplement: S2 Table — Summary statistics for the results obtained during measurements on the right and left leg, with eyes open and closed, and in the evening and morning, respectively. (DOCX) [file pone.0116695.s006.docx]

**Impaired postural control in healthy men at moderate altitude (1630 m and 2590 m). Data from a randomized trial.**

K. Stadelmann^1,2^, T. D. Latshang^3^, C. M. Lo Cascio^3^, R. A. Clark^5^, R. Huber^2,4^, M. Kohler^2,3^, P. Achermann^1,2^* and K. E. Bloch^2,3^*

**Table S2. Anterior-posterior sway amplitude at different altitudes, single leg tests**

| **Measur-**  **ement** | **Time** | **Eyes** | **490 m** | **1630 m Day 1** | **1630 m Day 2** | **2590 m Day 1** | **2590 m Day 2** | **P ANOVA Overall** |
| --- | --- | --- | --- | --- | --- | --- | --- | --- |
| **Right leg** | evening | open | 2.5 [2.2, 3.3] | 3.3 [2.9, 3.9] * | 3.3 [2.8, 3.7] * | 2.6 [2.3, 3.1] | 2.9 [2.3, 3.4] | <0.001 |
| **Left leg** | evening | open | 2.6 [2.2, 3.1] | 3.3 [2.8, 4.0] * | 3.3 [2.7, 3.8] * | 2.9 [2.3, 3.3] | 2.6 [2.4, 3.0] | <0.001 |
| **Right leg** | evening | closed | 6.5 [5.8, 8.6] | 6.4 [5.6, 8.4] | 6.9 [5.4, 8.7] | 6.9 [5.7, 8.2] | 6.9 [5.4, 8.5] | 0.714 |
| **Left leg** | evening | closed | 7.2 [5.7, 8.2] | 6.7 [5.4, 8.3] | 6.1 [5.2, 7.7] | 7.1 [6.0, 7.9] | 6.4 [5.5, 8.0] | 0.133 |
| **Right leg** | morning | open | 2.7 [2.3, 3.2] | 3.2 [2.7, 3.9] * | 3.2 [2.8, 4.0] * | 2.8 [2.2, 3.4] | 2.7 [2.3, 3.2] | <0.001 |
| **Left leg** | morning | open | 2.7 [2.3, 3.3] | 3.2 [2.7, 3.7] * | 3.3 [2.7, 3.9] * | 2.8 [2.3, 3.2] | 2.9 [2.4, 3.3] | <0.001 |
| **Right leg** | morning | closed | 7.1 [5.3, 8.1] | 6.6 [5.6, 8.3] | 6.5 [5.7, 8.6] | 7.4 [5.6, 8.7] | 7.2 [5.6, 8.5] | 0.416 |
| **Left leg** | morning | closed | 6.3 [5.5, 8.3] | 6.7 [5.8, 8.0] | 6.4 [5.4, 7.4] | 7.1 [6.3, 8.9] * | 7.2 [6.0, 9.0] | <0.001 |

Data are presented as median sway ampltitude in cm [25^th^, 75^th^ percentile]. Oxygen saturation values are the same as in table 2 of the main paper.

P ANOVA overall: Mixed model ANOVA with factor condition (490 m, 1630 m day 1, 1630 m day 2, 2590 m day 1, 2590 m day 2).

* p<0.0125 vs. to 490 m (Bonferroni correction), post-hoc Wilcoxon signed ranks.
