# Supplementary material for: Impaired Postural Control in Healthy Men at Moderate Altitude (1630 M and 2590 M): Data from a Randomized Trial
Source: PLoS One. 2015 Feb 27;10(2):e0116695. doi: 10.1371/journal.pone.0116695 (PMC4344242; doi:10.1371/journal.pone.0116695)
Supplement: S3 Table — Summary statistics for the results obtained during measurements on both legs, the right and left leg, with eyes open and closed, and in the evening and morning, respectively. (DOCX) [file pone.0116695.s007.docx]

**Impaired postural control in healthy men at moderate altitude (1630 m and 2590 m). Data from a randomized trial.**

K. Stadelmann^1,2^, T. D. Latshang^3^, C. M. Lo Cascio^3^, R. A. Clark^5^, R. Huber^2,4^, M. Kohler^2,3^, P. Achermann^1,2^* and K. E. Bloch^2,3^*

**Table S3: Medial-lateral sway amplitude at different altitudes**

| **Measurement** | **Time** | **Eyes** | **490 m** | **1630 m Day 1** | **1630 m Day 2** | **2590 m Day 1** | **2590 m Day 2** | **P**  **ANOVA**  **overall** |
| --- | --- | --- | --- | --- | --- | --- | --- | --- |
| **Both legs** | evening | open | 1.5 [1.3, 2.0] | 1.6 [1.3, 1.7] | 1.6 [1.4, 1.9] | 1.6 [1.3, 1.9] | 1.6 [1.3, 2.0] | 0.512 |
| **Right leg** | evening | open | 2.2 [2.0, 2.5] | 2.6 [2.4, 2.9] * | 2.4 [2.2, 2.9] * | 2.2 [2.0, 2.6] | 2.3 [2.0, 2.8] | <.001 |
| **Left leg** | evening | open | 2.3 [2.0, 2.7] | 2.6 [2.3, 2.9] * | 2.5 [2.1, 2.8] | 2.2 [1.9, 2.5] | 2.3 [1.8, 2.6] | <.001 |
| **Both legs** | evening | closed | 2.1 [1.6, 2.5] | 2.1 [1.6, 2.4] | 2.1 [1.5, 2.6] | 2.0 [1.5, 2.7] | 2.0 [1.5, 2.5] | 0.689 |
| **Right leg** | evening | closed | 5.0 [4.4, 5.5] | 4.8 [4.3, 5.6] | 4.6 [4.2, 5.3] | 4.7 [4.5, 5.2] | 4.7 [4.3, 5.2] | 0.366 |
| **Left leg** | evening | closed | 4.7 [4.2, 5.3] | 4.8 [4.3, 5.4] | 4.6 [4.1, 5.1] | 4.7 [4.3, 5.3] | 4.6 [4.2, 5.2] | 0.242 |
| **Both legs** | morning | open | 1.6 [1.3, 2.0] | 1.8 [1.4, 2.1] | 1.7 [1.4, 2.1] | 1.6 [1.3, 2.0] | 1.8 [1.3, 2.1] | 0.441 |
| **Right leg** | morning | open | 2.3 [2.0, 2.6] | 2.6 [2.3, 2.9] * | 2.5 [2.2, 2.8] * | 2.3 [2.0, 2.7] | 2.2 [1.9, 2.6] | <.001 |
| **Left leg** | morning | open | 2.2 [1.9, 2.5] | 2.4 [2.2, 2.7] * | 2.4 [2.1, 3.0] * | 2.2 [1.9, 2.6] | 2.2 [2.0, 2.7] | <.001 |
| **Both legs** | morning | closed | 2.0 [1.6, 2.5] | 2.1 [1.7, 3.0] | 2.1 [1.6, 2.5] | 2.1 [1.7, 2.8] | 2.0 [1.7, 2.6] | 0.620 |
| **Right leg** | morning | closed | 4.9 [4.4, 5.4] | 4.7 [4.2, 5.2] | 4.9 [4.3, 5.4] | 4.8 [4.3, 5.4] | 4.7 [4.3, 5.3] | 0.167 |
| **Left leg** | morning | closed | 4.7 [4.2, 5.3] | 4.7 [4.4, 5.1] | 4.7 [4.2, 5.1] | 4.8 [4.3, 5.3] | 4.7 [4.2, 5.3] | 0.670 |

Data are presented as median [25^th^, 75^th^ percentile].

P ANOVA overall: Mixed model ANOVA with factor condition (490 m, 1630 m day 1, 1630 m day 2, 2590 m day 1, 2590 m day 2).

* p<0.0125 compared to 490 m. Post-hoc Wilcoxon signed ranks test.
