# Supplementary material for: Impaired Postural Control in Healthy Men at Moderate Altitude (1630 M and 2590 M): Data from a Randomized Trial
Source: PLoS One. 2015 Feb 27;10(2):e0116695. doi: 10.1371/journal.pone.0116695 (PMC4344242; doi:10.1371/journal.pone.0116695)
Supplement: S4 Table — Results of univariate and multivariate logistic regression analysis performed on medial-lateral sway amplitude with altitude, pulse oximetry, altitude exposure sequence, consecutive number of tests, eyes open/closed, evening/morning, and age as independent variables. (DOCX) [file pone.0116695.s008.docx]

**Impaired postural control in healthy men at moderate altitude (1630 m and 2590 m). Data from a randomized trial.**

K. Stadelmann^1,2^, T. D. Latshang^3^, C. M. Lo Cascio^3^, R. A. Clark^5^, R. Huber^2,4^, M. Kohler^2,3^, P. Achermann^1,2^* and K. E. Bloch^2,3^*

**Table S4. Effect of altitude on medial-lateral sway: ordinal logistic regression analysis**

|  | Univariate | | | | Multivariate | | |
| --- | --- | --- | --- | --- | --- | --- | --- |
| Dependent variable: quintiles of medial-lateral sway | Odds ratio | | 95% CI | P | Odds | 95% CI | P |
| Altitude |  |  | |  |  |  |  |
| 1630 vs. 490 m | 1.09 | 0.84 to 1.41 | | 0.527 | 1.13 | 0.85 to 1.50 | 0.409 |
| 2590 vs. 490 m | 1.02 | 0.75 to 1.39 | | 0.900 | 1.00 | 0.71 to 1.40 | 0.994 |
| SpO2, % | 1.01 | 0.93 to 1.10 | | 0.751 |  |  |  |
| Altitude exposure sequence |  |  | |  |  |  |  |
| 2 vs. 1 | 0.85 | 0.36 to 2.01 | | 0.703 |  |  |  |
| 3 vs. 1 | 0.81 | 0.35 to 1.89 | | 0.626 |  |  |  |
| 4 vs. 1 | 0.60 | 0.23 to 1.55 | | 0.291 |  |  |  |
| Consecutive number of test days | 0.99 | 0.91 to 1.07 | | 0.810 |  |  |  |
| Eyes closed vs. eyes open | 3.57 | 2.65 to 4.80 | | <0.001 | 3.67 | 2.68 to 4.98 | <0.001 |
| Morning vs. evening test | 1.31 | 1.13 to 1.51 | | <0.001 | 1.32 | 1.12 to 1.55 | 0.001 |
| Age, y | 0.97 | 0.92 to 1.02 | | 0.190 | 0.96 | 0.92 to 1.02 | 0.192 |

Univariate and multivariate logistic regression was performed on quintiles of values of the medial-lateral sway amplitude with the lowest quintile corresponding to an odds ratio of 1. Altitude, age, and all variables with P<0.1 in univariate analysis were entered into the multivariate model. Altitude exposure sequence was: 1=2590-1630-490 m; 2=1630-2590-490m; 3=1630-2590-490 m; 4=2590-1630-490 m; consecutive number of test days was 1 to 5; eyes open and closed were coded as 1 and 2, respectively; morning and evening tests were coded as 1 and 2, respectively. CI=confidence interval.
